# Rac1 Is Required for Pathogenicity and Chm1-Dependent Conidiogenesis in Rice Fungal Pathogen *Magnaporthe grisea*

**DOI:** 10.1371/journal.ppat.1000202

**Published:** 2008-11-14

**Authors:** Jisheng Chen, Wu Zheng, Shiqin Zheng, Dongmei Zhang, Weijian Sang, Xiao Chen, Guangpu Li, Guodong Lu, Zonghua Wang

**Affiliations:** 1 Key Laboratory of Biopesticide and Chemistry Biology, Ministry of Education, Fujian Agriculture and Forestry University, Fuzhou, China; 2 The School of Life Sciences, Fujian Agriculture and Forestry University, Fuzhou, China; 3 Plant Protection College, Fujian Agriculture and Forestry University, Fuzhou, China; 4 Department of Biochemistry and Molecular Biology, University of Oklahoma Health Sciences Center, Oklahoma City, Oklahoma, United States of America; 5 The School of Animal Sciences, Fujian Agriculture and Forestry University, Fuzhou, China; University of Melbourne, Australia

## Abstract

Rac1 is a small GTPase involved in actin cytoskeleton organization and polarized cell growth in many organisms. In this study, we investigate the biological function of MgRac1, a Rac1 homolog in *Magnaporthe grisea*. The Mgrac1 deletion mutants are defective in conidial production. Among the few conidia generated, they are malformed and defective in appressorial formation and consequently lose pathogenicity. Genetic complementation with native MgRac1 fully recovers all these defective phenotypes. Consistently, expression of a dominant negative allele of MgRac1 exhibits the same defect as the deletion mutants, while expression of a constitutively active allele of MgRac1 can induce abnormally large conidia with defects in infection-related growth. Furthermore, we show the interactions between MgRac1 and its effectors, including the PAK kinase Chm1 and NADPH oxidases (Nox1 and Nox2), by the yeast two-hybrid assay. While the Nox proteins are important for pathogenicity, the MgRac1-Chm1 interaction is responsible for conidiogenesis. A constitutively active chm1 mutant, in which the Rac1-binding PBD domain is removed, fully restores conidiation of the Mgrac1 deletion mutants, but these conidia do not develop appressoria normally and are not pathogenic to rice plants. Our data suggest that the MgRac1-Chm1 pathway is responsible for conidiogenesis, but additional pathways, including the Nox pathway, are necessary for appressorial formation and pathogenicity.

## Author Summary

The fungus *Magnaporthe grisea* (*M. grisea*) is an important pathogen in plants and has a great impact on agriculture. Its infection of rice causes one of the most destructive diseases, the rice blast disease, around the world. *M. grisea* starts infection by producing conidia, which generate infectious structures and determine disease epidemics. However, the mechanism of conidial production is not well-understood. In this study, we have employed genetic and molecular techniques to silence the function of certain genes in *M. grisea* and found that the Rac1 gene is required for conidial production. Importantly, we have identified the mechanism for the Rac1 requirement in conidial production, which involves the interaction between Rac1 and its downstream effector Chm1. Furthermore, our study shows that the Rac1/Chm1-mediated conidiation is necessary but not sufficient for the pathogenicity of *M. grisea* in plants. Additional Rac1 effectors such as the Nox gene products are necessary for *M. grisea* to cause disease symptoms in rice and barley. Our study provides new insights into the mechanism of conidiation and pathogenicity of *M. grisea* during its infection in plants.

## Introduction

*Magnaporthe grisea* (*M. grisea*) is a good model organism to study plant pathogenic filamentous fungi [1],[2]. In addition, it is closely related to other prominent non-pathogenic model fungi, such as *Neurospora crassa* and *Aspergillus nidulans* [3]. The fungus infects many cereal crops such as rice, barley, and wheat, and causes rice blast, which is one of the most severe rice fungal diseases throughout the world [4],[5]. Under field condition, the infection starts with conidia landing on and attaching to a suitable surface of plant tissues with the help of the mucilage in spore tips [6]. Subsequently, the conidia germinate, form appressoria and invade the plant tissues. This is followed by invasive growth of the fungus [7],[8]. After successful colonization, many conidia are produced on the blast lesions and disseminated to new plant tissues and initiate a new infection cycle within 5–7 d. The severity of the rice blast disease epidemics is proportional to the quantity of spores produced in the lesion [9]. Therefore, many disease control strategies try to target conidiation, especially for the chemical control of the fungus [10]. However, the genetic basis and molecular mechanisms of conidiation are not well understood. Previous studies have identified several loci controlling conidiation [11]. Disruption of *con5* and *con6* abolishes conidial production. A series of other loci (*con1*, *con2*, *con4*, and *con7*), acting downstream of *con5* and *con6*, affect the development of conidia and sporulation. However, other than Con7p being shown as a transcriptional factor required for the transcription of several genes important for infection-related morphogenesis of the fungus [12], the other loci have yet to be characterized at the molecular level. Mgb1, a G-protein β-subunit, is involved in cAMP signaling that regulates conidiation, surface recognition, and appressorial formation. *mgb1* null mutation reduces conidiation, but does not abolish it [13]. In this regard, several other genes, *e.g.*, *chm1*, show similar functional phenotype to *mgb1* [14]. Therefore, the mechanism governing conidiation needs further characterization.

Rac1, a member of the Rho-family GTPases, exists in many eukaryotes [15], regulates actin cytoskeleton organization and cellular morphogenesis in higher eukaryotes [16]. In mammalian cells, the formation of actin-rich cell extensions termed lamellipodia is regulated by Rac [17]. In plants such as *Arabidopsis*, RAC/ROP GTPases regulate diverse processes ranging from cytoskeletal organization to hormone and stress responses [18]. Moreover, rice Rac homolog, OsRac1, plays a role in disease resistance by activating reactive oxygen intermediate (ROI) production and cell death [19].

Unlike the other Rho GTPases (CDC42, Rho), Rac orthologs are not found in yeast such as *Saccharomyces cerevisiae* and *Schizosaccharomyces pombe*. It is of great interest to study the function of Rac homologs in the development of filamentous fungi. In *Penicillium marneffei*, CflB, a Rac1 homolog, is involved in cellular polarization during its asexual development and hyphal growth but not involved in its yeast growth state at 37°C [20]. The *cflB* deletion mutants show cell division (septation) and growth defects in both vegetative hyphal and conidiophore cell types. In the human pathogen *Candida albicans*, Rac1 is not necessary for viability or serum-induced hyphal growth, but it is essential for filamentous growth when cells are embedded in a matrix [21]. In *Cryptococcus neoformans*, however, a Rac homolog controls haploid filamentation and high-temperature growth downstream of Ras1 [22]. In the pathogenic fungi of plants such as *Colletotrichum trifolii*, Rac1 functions downstream of Ras and can restore the hyphal morphology of dominant Ras mutants by regulating MAPK activation and intracellular reactive oxygen species (ROS) generation [23]. In another phytopathogenic fungus *Ustilago maydis*, Rac1 is required for pathogenicity as well as proper cellular morphology and hyphal growth [24]. Recently, Rolke and Tudzynski [25] reported that Rac1 interacts with Cla4, and regulates the polarity, development and pathogenicity in *Claviceps purpurea*. Thus, Rac GTPases play an important role in fungal development.

In the current study, we investigate the function of MgRac1, a Rac1 homolog in *M. grisea*, and show that MgRac1, is essential for conidiogenesis, and contributes to the formation of appressorium and pathogenicity of *M. grisea* through activating its downstream effectors: the PAK kinase Chm1 and NADPH oxidases.

## Results

### MgRac1 is a Rac1 homolog in *M. grisea*

The *M. grisea* genome encodes a Rac homolog in the locus MGG_02731.5 [2]. It contains five GTP/GDP binding or hydrolysis motifs (G1 through G5) characteristic of Rho-family small GTPases. The conserved G4 motif has a TKLD sequence characteristic of Rac, and is distinct from that found in Rho (T/NKXD) and Cdc42 (TQXD) [16]. We hereafter named it as MgRac1 (*Magnaporthe grisea* Rac1). The multiple alignment analysis showed that MgRac1 is highly homologous to Rac1 homologs from other filamentous fungi, including the plant pathogens *Colletotrichum trifolii* (CtRac1, AAP89013, 94% identity), *Fusarium graminearum* (FgRac1, EAA72031, 93% identity), and *Stagonospora nodorum* (SnRacA, SNOG_00327.1, 88% identity).

### MgRac1 is essential for conidiogenesis

**Figure 1:**
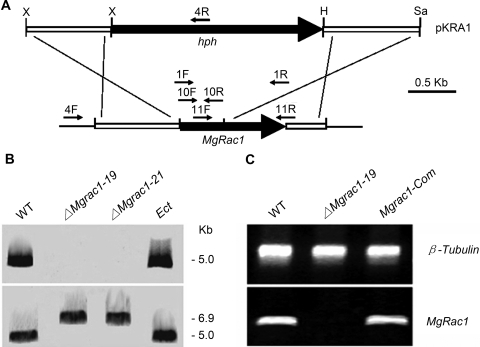
Construction and confirmation of the *Mgrac1* deletion mutant. (A) Restriction map of the MgRac1 genomic region and deletion construct pKRA1. Thick arrows indicate orientations of the MgRac1 and hygromycin phosphotransferase (*hph*) genes. The restriction enzymes are abbreviated as X (*Xho*I), H (*Hin*dIII), and Sa (*Sac*I). The Mgrac1 deletion construct pKRA1 contained the homologous sequences flanking the *hph* gene to replace the first 525-bp of the MgRac1 ORF. Primers 4F and 4R (Table 4) were used for screening the Mgrac1 deletion mutants. (B) Total genomic DNA samples (5 µg per lane) isolated from WT (wild-type strain 70-15), *ΔMgrac1-19* (Mgrac1 deletion mutant), *ΔMgrac1-21* (Mgrac1 deletion mutant), and *Ect* (Ectopic transformant) were digested with *Pst*I and subjected to Southern blot analysis. The first probe, a 525-bp PCR fragment amplified from the genomic DNA of wild-type strain 70-15 using primers 10F and 10R (Table 4), is exactly the MgRac1 fragment replaced by the 2.6-kb hph gene and detects only the WT and *Ect* (top panel). The same blot was then stripped and re-hybridized with a 673-bp probe amplified from the 70-15 genomic DNA by primers 11F and 11R (Table 4) and this probe detects both WT and mutant DNA fragments, with the two deletion mutants showing a larger fragment due to the gene replacement (bottom panel). (C) Total RNA samples (approximately 1 µg per reaction) isolated from mycelia of WT, *ΔMgrac1-19* and *Mgrac1-Com* (MgRac1 complementary transformant) were subjected to RT-PCR using MgRac1 gene-specific primers 1F and 1R (Table 4). The RT-PCR product is a 600-bp fragment in WT and *Mgrac1-Com* as predicted, but is missing in the deletion mutant *ΔMgrac1-19*.

To study the function of MgRac1 in the fungus, we first generated *Mgrac1* deletion mutants by replacement of the *MgRac1* ORF with a selective marker [the bacterial phosphotransferase (hph) gene], through transformation of protoplasts of the wild-type *M. grisea* strain 70-15 with the deletion construct pKRA1 (Figure 1A). Deletion transformants were screened by growing on selection media supplemented with hygromycin and by PCR verification of genomic DNA of the transformants. The putative deletion mutants were further confirmed by Southern blotting (Figure 1B) and RT-PCR (Figure 1C). Two deletion mutants *ΔMgrac1-19*, *ΔMgrac1-21*, and one ectopic transformant (*Ect*), which had the marker inserted into regions other than the MgRac1 gene, were selected for further analysis in this study. Furthermore, we constructed a complementation strain *Mgrac1-Com* by reintroducing the genome DNA sequence including a 1.2-kb promoter region and the ORF of *MgRac1*.

**Figure 2:**
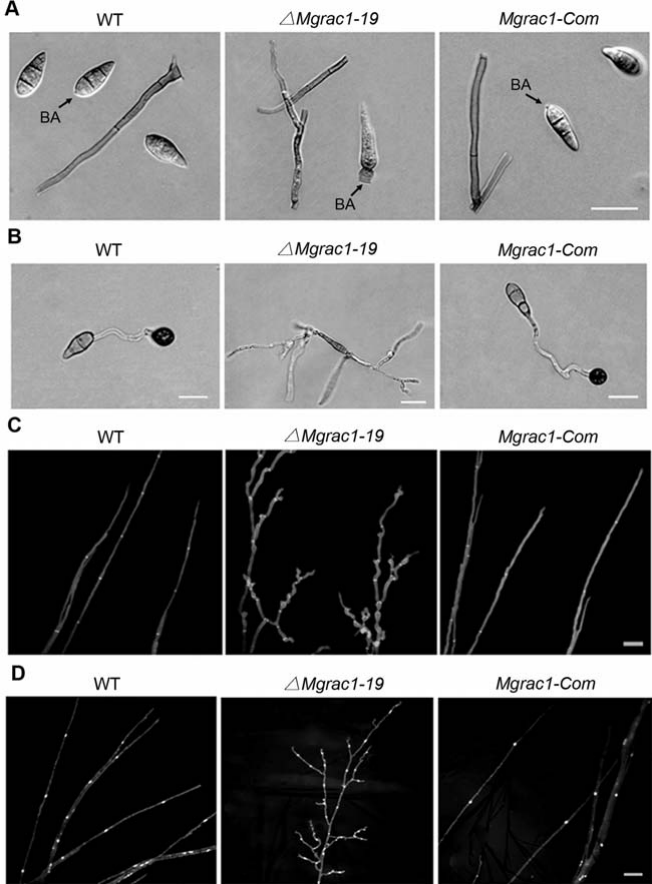
Abnormal conidial morphology, appressorial formation, and hyphal branching in the Mgrac1 deletion mutant. (A) Differential interference contrast (DIC) microscopy of conidia cultured on an oatmeal agar plate at day 10 after incubation. BA = basal appendage where conidia attach to conidiophores. Bar = 20 µm. (B) Conidia incubated on the surface of artificial hydrophobic Gelbond films as described in Materials and Methods. Bar = 20 µm. (C) Branching patterns of mycelia on complete media plates at day 3 after incubation. Frequent branching occurs at the terminal mycelia of *ΔMgrac1-19*. Calcofluor staining of mycelia is used to show the distance of septa. Bar = 20 µm. (D) DAPI staining of mycelia to show the localization of nuclei. Bar = 20 µm.

Conidiation of the wild-type strain (70-15), *Mgrac1* deletion mutants (*ΔMgrac1-19* and *ΔMgrac1-21*) and *MgRac1* complement strain (*Mgrac1-Com*) on 10-day-old oatmeal agar cultures were determined. The most striking finding was that conidiation was dramatically reduced by 3 orders of magnitude in *Mgrac1* deletion mutants (Table 1). In contrast, the wild-type strain 70-15 and the complement strain were normal in sporulation under the same conditions (Table 1). Of the few conidia that formed in *ΔMgrac1-19* and *ΔMgrac1-21*, most exhibited abnormal, elongated morphology (Figure 2A), which was also observed in a T-DNA insertion line by Jeon [26]. The constriction at the base of the malformed conidia was incompletely formed, and consequently the conidia could not detach normally from the conidiophore as in wild type (Figure 2A). As a result, a basal appendage (BA, Figure 2A) remained attached, similar to that observed in the *chm1* deletion mutant [14]. The data indicate that MgRac1 is essential for the conidiogenesis of *M. grisea*.

**Table 1:** Phenotypic analysis of *MgRac1* mutants.

Strain	Saprophytic growth (mm/day)^a^	Conidiation (*10^4^)^b^	Penetration (%)^c^	Lesions on 5-cm-long rice leaf tip^d^
**70-15**	6.18±0.38^e^	297.82±16.44	70.05±9.03	65.45±4.76
**Guy11**	5.67±0.62	395.83±16.71	75.64±7.32	72.67±5.11
*Δ* ***Mgrac1-19***	5.01±0.17	0.07±0.03	0	0
***Mgrac1-Com***	5.63±0.26	283.32±28.83	71.12±6.59	61.25±8.18
***MgRac1-CA***	6.27±0.53	154.96±22.74	3.66±1.42	11.75±3.14
***MgRac1-DN***	5.28±0.33	0.05±0.02	0	0
***PCG33***	5.75±0.02	308.9±12.79	65.76±6.85	63.83±4.65
***PCA19***	5.77±0.05	265.07±10.43	5.14±2.55	0
***chm1***	3.93±0.66	0.55±0.25	0	0
***RCC3*** f	5.34±0.73	5.35±0.83	0	0
***RCC6*** f	5.46±0.04	5.4±0.74	0	0

a Diameter of hyphal radii at day 8 after incubation on complete medium agar plates at room temperature.

b Number of conidia harvested from a 9 cm oatmeal plate at day 10 after incubation at room temperature.

c Percentage of penetration over total number of appressoria at 24 h post-inoculation on onion epidermis.

d Lesion number 5 days after inoculation.

e Data in all columns are the means of three independent experiments with standard deviations.

f Two independent transformants expressing constitutively active MgRac1-CA in the chm1 null background.

We next examined the *MgRac1* gene expression profiles at different growth stages by quantitative real-time PCR. The results showed much higher expression level of *MgRac1* in conidium than in mycelium, germ tube and appressorium (Table 2), consistent with its important role in conidiation and conidial morphology. Interestingly, the *Mgrac1* deletion mutants could still form conidiophores (Figure 2A), even though conidial production was severely reduced.

**Table 2:** Real-time RT-PCR quantification of *MgRac1* expression in *M. grisea*.

RNA source	*MgRac1* C_T_ a	β-tubulin C_T_	Normalized *MgRac1* level relative to β-tubulin^b^
**Wild-type mycelium**	24.43±0.11	25.42±0.13	1.00 (0.96–1.09)^c^
**Wild-type conidium**	21.65±0.06	25.63±0.09	7.88 (7.84–8.05)
**Wild-type germ tube**	26.21±0.05	25.58±0.10	0.33 (0.29–0.35)
**Wild-type appressorium**	22.64±0.06	25.78±0.08	4.41 (4.35–4.59)

a Cycle number at which the fluorescence crossed the threshold. Mean and standard deviation were calculated with data from three replicates.

b Relative quantity of MgRac1 at different developmental stages of the wild-type strain 70-15.

c The mean and range of three replicates.

Although the few conidia from the *Mgrac1* deletion mutants had abnormal morphology, over 90% of them germinated after 24 h of incubation at room temperature (data not shown). However, appressorial formation from these mutant conidia was completely blocked on the hydrophobic side of GelBond membranes by 24 h (Figure 2B). In contrast, over 95% of germ tubes formed appressoria in the wild-type strain 70-15 and *MgRac1* complement strain *Mgrac1-Com* under the same conditions (Figure 2B). Even after prolonged incubation (over 72 h), no appressorium was observed in the *Mgrac1* deletion mutants.

Frequent branching and curly tips were observed at the terminal mycelia of the *Mgrac1* deletion mutant (*ΔMgrac1-19*). However, Calcofluor staining of cell walls of mycelia showed that the septa were normal except for shorter intervals (Figure 2C). Like 70-15, the *ΔMgrac1-19* mutant had one nucleus in each hyphal compartment, suggesting that nuclear division and cytokinesis were normal in the *Mgrac1* mutant (Figure 2D). These data indicate that *MgRac1* is dispensable for septal formation in the fungus *M. grisea*. Furthermore, we compared radial hyphal growth of the wild-type strain (70-15), *Mgrac1* deletion mutants (*ΔMgrac1-19*) and *MgRac1* complement strain (*Mgrac1-Com*) on CM agar media. The *Mgrac1* deletion mutants produced typical grayish *M. grisea* mycelia. But the colonies of the *Mgrac1* mutants were coralline-like and slightly smaller, due to slower growth rate (Table 1).

### *Mgrac1* deletion mutants are nonpathogenic

**Figure 3:**
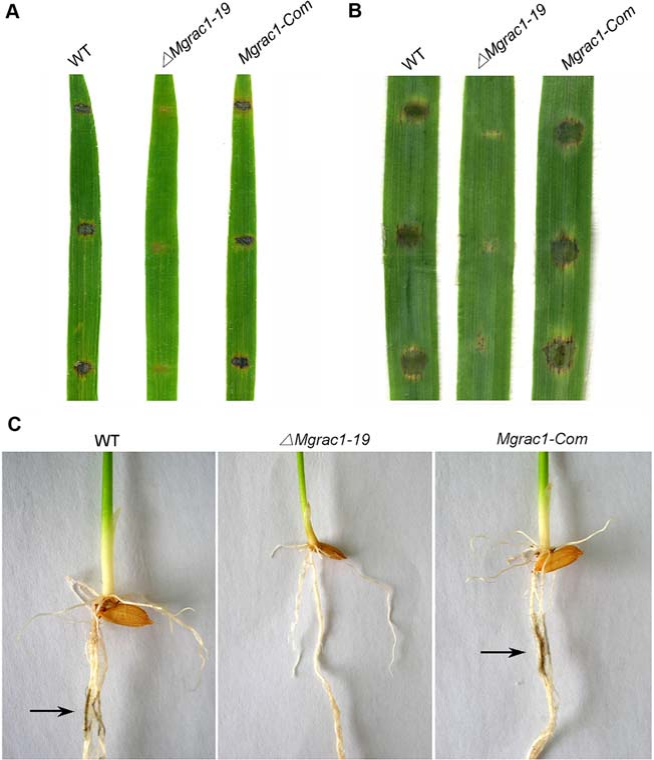
The Mgrac1 deletion mutant is nonpathogenic. (A) Disease symptoms on the wounded leaf tissues of rice inoculated by mycelial plugs from WT (70-15), *ΔMgrac1-19*, and *Mgrac1-Com*. Typical leaves were photographed 5 days after inoculation. (B) Disease symptoms on the wounded leaf tissues of barley inoculated by mycelial plugs from WT, *ΔMgrac1-19*, and *Mgrac1-Com*. Typical leaves were photographed 4 days after inoculation. (C) Blast symptoms on rice roots. Arrows show necrotic lesions.

Because the *Mgrac1* deletion mutants hardly produced any conidia, and were defective in appressorial formation, we used mycelia plugs of the deletion mutants to inoculate wounded rice leaves (Figure 3A), wounded barley leaves (Figure 3B), and rice roots (Figure 3C). No disease symptoms developed either on wounded leaves and rice roots. In contrast, the wild-type strain (70-15), and *MgRac1* complement strain (*Mgrac1-Com*) caused typical rice blast lesions in the same tissues at 4–5 days post-inoculation (dpi) (Figure 3). The data indicate that *Mgrac1* deletion mutants are nonpathogenic, and that MgRac1 GTPase is essential for the pathogenicity of *M. grisea*.

### Ectopic expression of dominant negative and constitutively active *MgRac1* alleles results in defects in conidiogenesis and pathogenicity

To further investigate the function of MgRac1 GTPase, we constructed both a dominant negative form of MgRac1 by substituting aspartic acid at position 128 with alanine (D128A, DN), and a constitutively active form of MgRac1 by substituting glycine at position 17 with valine (G17V, CA). After transforming the protoplasts of wild-type strain 70-15 with *MgRac1-DN* and *MgRac1-CA*, respectively, positive transformants were identified by Southern blot analysis and further characterized as described above. Real-time PCR analysis indicated that there was a 8-fold and 20-fold increase of *Rac1* expression in vegetative hyphae of *MgRac1-DN* and *MgRac1-CA* mutants compared with the wild-type strain 70-15, respectively (Table 3), suggesting that the transformants expressed the expected dominant alleles of MgRac1.

**Table 3:** Real-time RT-PCR quantification of the transcripts of *Rac1*, *Cdc42*, *Chm1*, *Nox1*, and *Nox2* in different *Magnaporthe grisea* mutants.

Mutant strain	*Rac1* a	*Cdc42*	*Chm1*	*Nox1*	*Nox2*
*Δ* ***Mgrac1-19***	0	1.61±0.14	0	0.16±0.03	0.23±0.07
***MgRac1-CA***	20.15±1.54	0.81±0.02	7.32±0.84	5.33±0.68	4.16±0.63
***MgRac1-DN***	8.33±1.38	1.44±0.23	0.23±0.03	0.19±0.05	0.79±0.12
***MgRac1-OE***	23.88±3.01	0.22±0.03	2.67±0.35	3.32±0.58	3.21±0.63

a Relative quantity of the indicated transcripts in the mutant strains, relative to that in the wild-type strain 70-15. A value of greater than 1 indicates increased expression, while a value of smaller than 1 indicates decreased expression. Mean and standard deviation were calculated with the data from three replicates.

**Figure 4:**
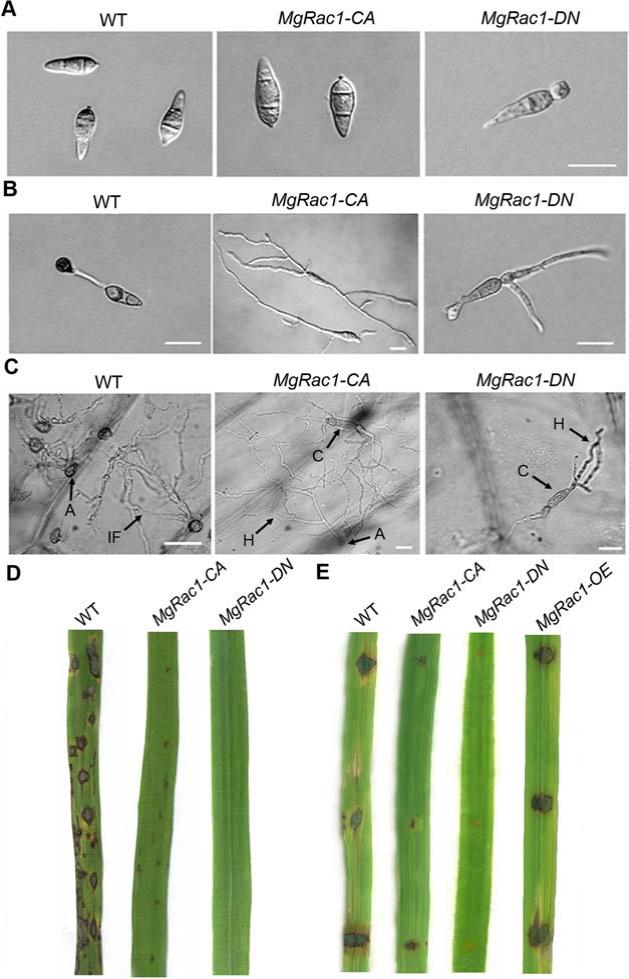
Abnormal conidial morphology, appressorial formation, and pathogenicity of the MgRac1 dominant mutants. (A) Differential interference contrast (DIC) microscopy of conidia collected from WT (70-15), *MgRac1-CA* (constitutively active mutant), and *MgRac1-DN* (dominant negative mutant), as indicated. Bar = 20 µm. (B) Conidial suspensions of *MgRac1-CA and MgRac1-DN* were applied on the hydrophobic side of Gelbond film and examined with DIC microscopy. Bar = 20 µm. (C) Conidial suspensions (about 1,000 conidia in 20 µl) of 70-15 and MgRac1 mutants were inoculated on strips of onion epidermis. Infectious hyphae were photographed 2 days after inoculation with DIC microscopy. A = appressorium, C = conidium, H = hypha, IF = infectious hypha. Bar = 20 µm. (D) Leaves of rice cultivar CO39 were sprayed with conidial suspensions (1×10^5^ conidia/ml) from WT, *MgRac1-CA*, and *MgRac1-DN*. Typical leaves were photographed at 7 days after inoculation. (E) Disease symptoms on the wounded leaf tissues of rice inoculated with conidia (5×10^4^ conidia/ml) from WT and MgRac1 mutants, as indicated. And unwounded rice leaf tissue was inoculated with the mutant of *MgRac1-OE*. Typical leaves were photographed 5 days after inoculation.

Like the *Mgrac1* deletion mutants, the *MgRac1-DN* mutant produced malformed conidia (Figure 4A), failed to develop appressoria after germination (Figure 4B) and failed to penetrate the onion epidermis (Figure 4C), and consequently lost pathogenicity on rice either by spraying (Figure 4D) or inoculating wounded leaves (Figure 4E). *MgRac1-CA* produced only half amount of conidia (Table 1) and they exhibited small but significant (p<0.01) increase in size (Figure 4A) in comparison to the conidia of wild-type strain 70-15 based on the one way ANOVA analysis. The length and width of *MgRac1-CA* conidia were 22.87±0.11 µm and 10.11±0.15 µm, while those of 70-15 were 21.25±0.07 µm and 9.13±0.03 µm, respectively, in which the mean values and standard deviations were calculated on measurements of 50 conidia per replicate for 3 replicates in 5 independent experiments by using program SPSS V13.0. However, there was no change in the length and width ratio. The conidia from *MgRac1-CA* were able to adhere to the surface and germinate, but failed to form appressoria on hydrophobic sides of Gelbond membrane (Figure 4B), and only a few appressoria developed on onion epidermis after 48 hours (Figure 4C). Under the same conditions, the conidia of the wild-type strain 70-15 developed normal and well-melanized appressoria (Figure 4B), which penetrated onion epidermis successfully and developed infectious hyphae (Figure 4C). The *MgRac1-CA* strain failed to cause disease on rice seedlings (Figure 4D), and wounded rice leaves (Figure 4E), probably due to the defect in appressorial development and infectious growth. Although there were some small brown lesions when sprayed with conidial suspensions, these lesions did not produce any conidia even after prolonged incubation in high moisture after detachment for two days. In contrast, the wild-type strain efficiently generated susceptible lesions that all produced conidia after incubation (Figure 4D and 4E). The data indicate that although *MgRac1-CA* shows opposite effect on conidiogenesis in comparison to *MgRac1-DN*, their conidia are nonfunctional and defective in appressorial formation and pathogenicity. To confirm that the phenotypes of DN and CA mutants shown in Figure 4 are indeed due to their constitutively active and dominant negative mutations, as opposed to the elevation in Rac1 protein levels, we constructed over-expression (OE) mutant of MgRac1 and compared their phenotypes. Real-time PCR analysis indicated that there was a 23.88±3.01 fold increase of Rac1 expression in vegetative hyphae of the *MgRac1-OE* mutant, which also affected expression level of *Cdc42*, *Chm1*, *Nox1* and *Nox2* compared with that of the wild-type strain (Table 3). However, the over-expression of *MgRac1* had no obvious effect on conidiogenesis (data not shown) and pathogenicity (Figure 4E) of *M. grisea*, which indicated that the phenotypes of *MgRac1-DN* and *MgRac1-CA* mutants are due to their dominant mutations, rather than the elevation in Rac1 expression levels.

**Figure 5:**
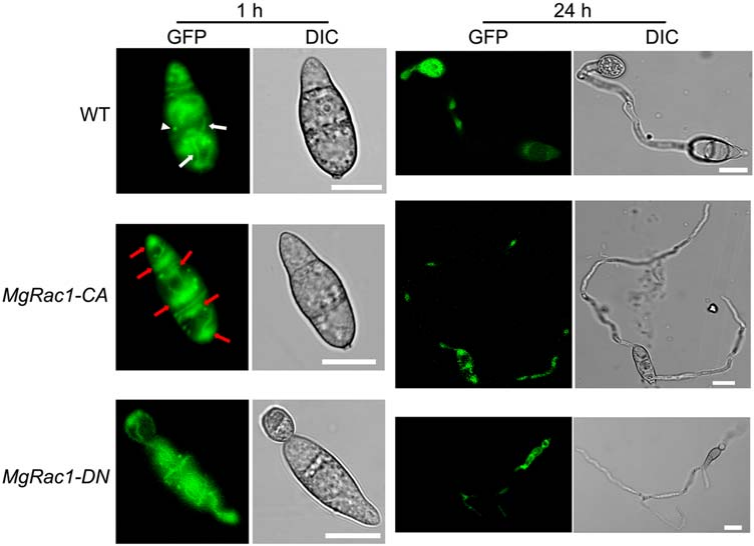
Cellular localization of tropomyosin-GFP in *MgRac1-CA and MgRac1-DN* mutants. Conidia expressing heterologous tropomyosin-GFP from WT (wild-type strain Guy11), *MgRac1-CA*, and *MgRac1-DN* were incubated on Gelbond films at 1 h and 24 h and observed by confocal fluorescence microscopy. Arrowhead indicates TpmA-GFP-labeled actin spot, white arrows indicate actin filaments, and red arrows indicate the areas where actin structures accumulate. Bar = 10 µm.

Next we examined the effects of *MgRac1-CA* and *MgRac1-DN* on actin organization in condia, since Rac1 was shown to play an important role in actin organization in other organisms [27],[28]. In this case, we employed a heterologous tropomyosin-GFP (*TpmA-GFP*) fusion protein that was previously shown to bind and label actin cables in the filamentous fungus *Aspergillus nidulans* [29]. This TpmA-GFP cassette was transferred to *M. grisea* at the background of the wild-type strain Guy11, which had two copies of *TpmA-GFP* (provided by Dr. Talbot), and the protoplasts were then transformed with *MgRac1-CA* and *MgRac1-DN*, respectively. Conidia were collected and examined by Zeiss LSM 510 confocal microscopy at 1 h and 24 h post-incubation. Strong GFP fluorescence was detected in the cytoplasm. At 1 h after the germination began, the *TpmA-GFP*-labeled actin structures were mostly distributed in the cytoplasm with some discernable actin filaments in the wild-type strain (WT) (Figure 5). The actin filaments were sometimes found attached to bright *TpmA-GFP*-labeled spots (Figure 5), which resembled the actin bodies in quiescent yeast cells returning to growth [30]. In the *MgRac1-CA* mutant, however, the labeled actin structures accumulated at the polarization sites and showed bipolar distribution in each of the three cells in the conidium, with actin filaments more evident than in WT (Figure 5). In the *MgRac1-DN* mutant, some actin structures also accumulated at both ends of the conidium but most *TpmA-GFP*-labeled actin filaments appeared abnormally straight and striated in the middle of the cytoplasm (Figure 5), which could contribute to its elongated morphology. After 24 h incubation, most of the *TpmA-GFP*-labeled actin structures in WT moved from the conidium to the appressorium, but they remained in the conidia of the *MgRac1* mutants (Figure 5). The data suggest that in the *MgRac1-DN* and *MgRac1-CA* mutants, actin is not properly organized and cannot be mobilized for the formation of appressorium and pathogenicity.

### MgRac1 physically interacts with Chm1 via its PBD domain and genetically acts upstream of Chm1 to activate conidiogenesis

To understand the mechanism of MgRac1-mediated conidiogenesis and pathogenicity in *M. grisea*, we further investigated functional relationship of MgRac1 with Chm1, which is a Cla4 homolog of the baker yeast *Saccharomyces cerevisiae*. Cla4 is a p21-activated kinase (PAK), which contains a p21-Rho-binding domain (PBD) and a kinase domain. PAK is known to directly transmit signal from Rac/Cdc42 GTPase by acting as a Rac/Cdc42 effector in yeast [31]. The PBD domain is also known as the CRIB domain (Cdc42/Rac-interactive-binding domain) and responsible for interaction with the active form of Rac/Cdc42 [32]. In *chm1* deletion mutants of *M. grisea*, colony growth rate and conidiation are dramatically reduced and of the few conidia produced, most exhibited abnormal morphology and function [14], similar to the phenotype of our *Mgrac1* deletion mutants. Moreover, the hyper-branching phenotype in the growing hyphae of the *chm1* deletion mutants is the same as that of the *Mgrac1* deletion mutants. Thus we examined the relationship between MgRac1 and Chm1. Real-time PCR analysis indicated that there was a 7-fold increase of *Chm1* expression in the *MgRac1-CA* mutant and a decrease in the *MgRac1-DN* mutant (Table 3). When *MgRac1* was deleted, *Chm1* transcript was almost undetectable relative to the wild-type 70-15 transcript (Table 3).

**Figure 6:**
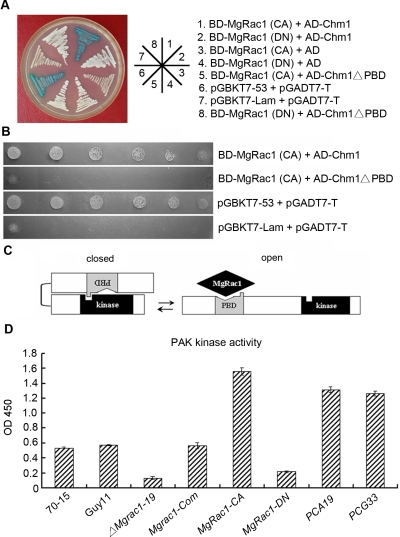
The interaction between MgRac1 and Chm1 or Chm1*Δ* *PBD* and PAK activity assay. (A) Yeast two-hybrid assay with MgRac1-CA or MgRac1-DN as the bait and Chm1 or Chm1*ΔPBD* as the prey. Yeast transformants grown on the SD-Leu-Trp plates were assayed for β-galactosidase activity. The interaction of pGBKT7-53 and pGADT7-T was used as the positive control. The interaction of BD-MgRac1(CA) or BD-MgRac1(DN) and AD (pGADT7) was used as the negative control to rule out self-activation. (B) The indicated yeast transformants diluted to specified concentrations (cell/ml) were plated onto SD-Ade-Leu-Trp-His to examine the *HIS3* reporter gene expression in the yeast two-hybrid assay. The interaction of pGBKT7-Lam and pGADT7-T was used as the negative control. (C) Model of Chm1 activation and its auto-inhibition by the PBD domain. It involves transition between low-activity (closed) and high-activity (open) conformations. The PBD domain (grey) contains domains that bind MgRac1 and the PAK kinase domain, as indicated. (D) PAK kinase assay showing correlation of MgRac1 and PAK activity in the hyphae of WT and mutants. Total protein preparations were subjected to the kinase assay, which used the HTScan PAK1 kinase assay kit for direct ELISA detection of the product at the absorbance of 450 nm. Means and standard deviation calculated from three replicates were shown on the bar chart.

We further investigated whether Chm1 can act as a MgRac1 effector to control conidiogenesis and pathogenicity. If Chm1 is MgRac1 effector, it is expected to physically interact with activated GTP-bound MgRac1 and genetically act downstream of MgRac1. We used the yeast two-hybrid assay to test whether the constitutively active and the dominant negative forms of MgRac1 can interact with either full-length Chm1 or the Chm1ΔPBD mutant in which the PBD domain is removed. The results showed that Chm1 was able to interact with the constitutively active, but not the dominant negative form of MgRac1 (Figure 6A and 6B), indicating that Chm1 is an effector of MgRac1. The results also showed that the PBD domain of Chm1 was responsible for this interaction, since deletion of the PBD domain abolished the Chm1-MgRac1 interaction (Figure 6A and 6B).

We next tested whether Chm1 genetically and functionally acts downstream of MgRac1 in conidiogenesis. As a homolog of PAK kinase, the PBD domain of Chm1 is expected to act as an auto-inhibitory domain to suppress the kinase activity [32]. Upon binding to activated Rac1, the PBD domain is released leading to Chm1 activation (Figure 6C). Thus removal of the PBD domain should make the Chm1 PAK kinase constitutively active. To confirm this, a *CHM1ΔPBD* construct was made under the control of its native promoter and used for transformation of the *Mgrac1* deletion mutant and the wild-type strain Guy11 to generate the double mutants *PCA19* and *PCG33*, respectively. Northern blot analysis confirmed the expression of *CHM1ΔPBD* transcript in the double mutants, which was smaller than the transcript of wild-type *CHM1* (data not shown).

We determined the PAK kinase activity in these mutants. Total protein of vegetative hyphae was subjected to in vitro PAK kinase assay using HTScan PAK1 kinase assay kit. As shown in Figure 6D, PAK kinase activity in both *PCA19* and *PCG33* mutants was increased by more than two-fold over endogenous PAK activity, indicating that the expressed *CHM1ΔPBD* was active. In a series of control experiments, we found that the *ΔMgrac1-19* and *MgRac1-DN* mutants significantly reduced the PAK activity relative to the WT strains. In contrast, the constitutively active *MgRac1-CA* mutant greatly increased the PAK kinase activity (Figure 6D). These data demonstrate that *MgRac1-DN* and *MgRac1-CA* are effective dominant negative and positive mutants, respectively.

**Figure 7:**
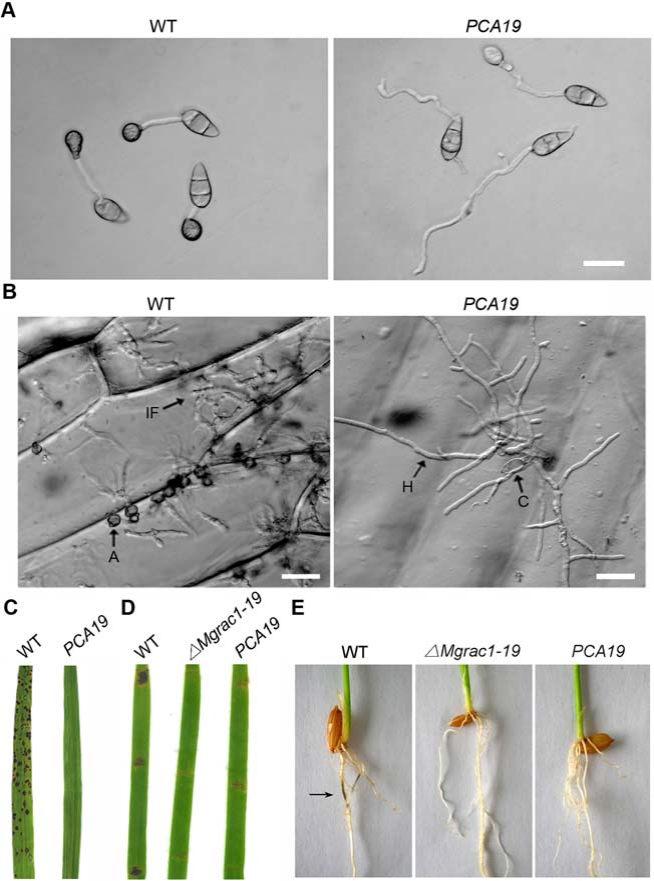
Chm1*Δ* *PBD* rescues conidiation in *Mgrac1* deletion mutants. (A) DIC microscopy of conidia of WT (70-15) and *PCA19* (Chm1*ΔPBD* expression in the Mgrac1 deletion mutant) collected after incubation the hydrophobic Gelbond film surface. Bar = 20 µm. (B) Conidia suspensions (about 1,000 in 20 µl) of WT and *PCA19* were inoculated on strips of onion epidermis. Infectious hyphae were examined at 1 day post-inoculation with DIC microscopy. A = appressorium, C = conidium, H = hypha, IF = infectious hypha. Bar = 20 µm. (C) Leaves of rice cultivar CO39 were sprayed with conidial suspensions (1×10^5^ conidia/ml) from WT and *PCA19*. Typical leaves were photographed 7 days after inoculation. (D) Disease symptoms on the wounded leaf tissues of rice inoculated with mycelial plugs from WT, *ΔMgrac1-19*, and *PCA19*. Typical leaves were photographed 5 days after inoculation. (E) Blast symptoms on rice roots. Arrow indicates necrotic lesions.

We then focused on the double mutants to investigate the genetic relationship of MgRac1 and Chm1. Indeed, the double mutant *PCA19* recovered in conidiation, produced normal conidia both in morphology (Figure 7A) and in quantity like the wild-type strain (Table 1). In addition, the *PCG33* mutant showed no obvious defect in morphology and pathogenicity (Table 1). The data indicate that the constitutively active *CHM1ΔPBD* can fully rescue the conidiogenesis defect in the *Mgrac1* deletion mutant, and that MgRac1 genetically acts upstream of Chm1 to activate the conidiogenesis pathway. However, despite normal production and morphology, the conidia of *PCA19* were not functional in terms of further appressorial development and pathogenicity (Figure 7). Although the constitutively active *CHM1ΔPBD* mutant rescued the condiation defect of the *Mgrac1* deletion mutant, the constitutively active *MgRac1-CA* mutant did not rescue the defect of the *chm1* deletion mutant (*RCC3* and *RCC6* in Table 1). The data further support the assumption that Chm1 is a downstream effector of MgRac1 to control conidiogenesis, but additional effectors of MgRac1 are required for pathogenicity of the fungus *M. grisea*.

### NADPH oxidases Nox1 and Nox2 are MgRac1 effectors required for pathogenecity but not for conidiogenesis

*M. grisea* genome contains two superoxide-generating NADPH oxidase genes, *Nox1* and *Nox2*. The *Nox* proteins were described as Rac1 effectors in other organisms [33] and it was shown genetically that each was independently required for the pathogenicity of *M. grisea* [34]. Thus we further investigated if MgRac1 physically interacts with Nox1 and Nox2 and if the interactions play a role in the conidiogenesis and pathogenicity of *M. grisea*. We first conducted real-time PCR analysis to examine the relationship between MgRac1 and *Nox* gene expression. There was a 5-fold increase in the levels of *Nox1* and *Nox2* transcripts in the *MgRac1-CA* mutant over the wild-type strain 70-15 (Table 3). In contrast, there was a 6-fold decrease in the levels of *Nox1* and *Nox2* transcripts in the *ΔMgrac1-19* and *MgRac1-DN* mutants (Table 3). This correlation in gene expression between MgRac1 and Nox is similar to that between MgRac1 and Chm1 and suggests that the NADPH oxidases are also potential MgRac1 effectors in *M. grisea*.

**Figure 8:**
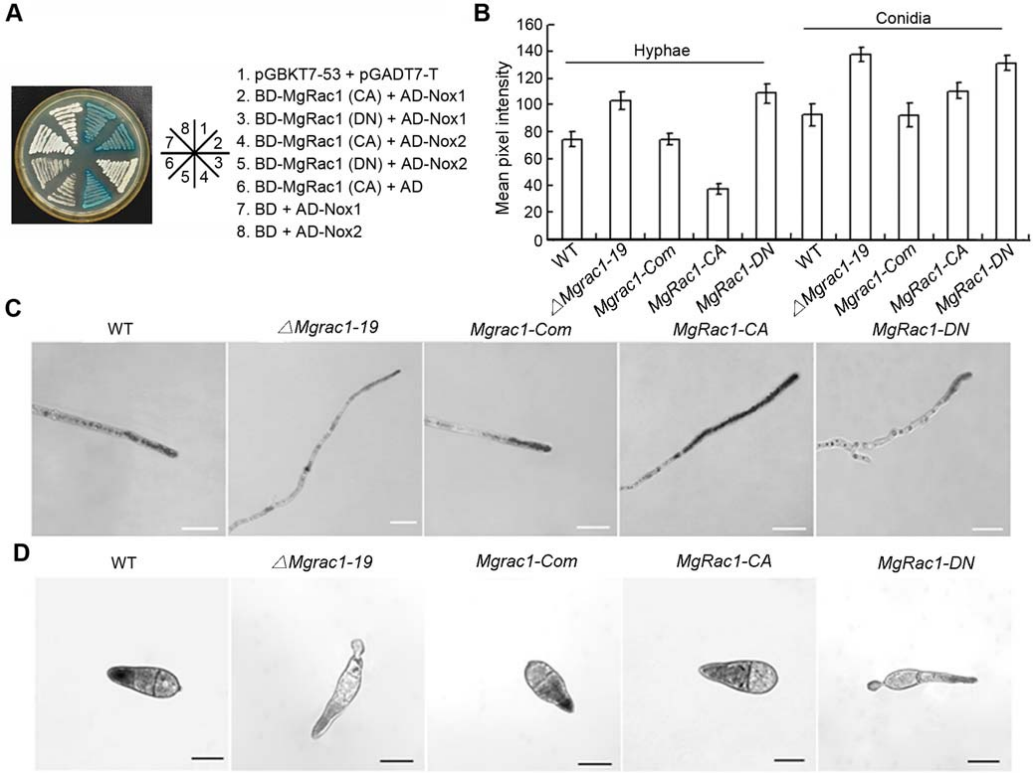
The interaction between MgRac1 and Nox1/Nox2 and superoxide production in MgRac1 mutants. (A) Yeast two-hybrid assay with *MgRac1-CA* or *MgRac1-DN* as the bait and Nox1 or Nox2 as the prey. Yeast transformants grown on the SD-Leu-Trp plates were assayed for β-galactosidase activity. The interaction of pGBKT7-53 and pGADT7-T was used as the positive control. The interaction of BD-MgRac1(CA) and AD (pGADT7) as well as BD (pGBKT7) and AD-Nox1/2 were used as negative controls to rule out self-activation. (B) Bar chart showing mean pixel intensity in hyphal tips and conidia of WT (70-15) and MgRac1 mutants, which quantifies the results in (C) and (D). Increased staining by NBT means reduced pixel intensity. Error bar means standard deviation based on the data of three independent experiments. (C) Detection of superoxide production by 0.6 mM NBT staining in the hyphal tips of WT and MgRac1 mutants. Bar = 10 µm. (D) Detection of superoxide production by 0.3 mM NBT staining in the conidia of WT and MgRac1 mutants. Bar = 10 µm.

We then tested whether Nox1 and Nox2 can physically interact with MgRac1 and genetically act downstream of MgRac1 as effectors to control conidiogenesis and pathogenicity. We used the yeast two-hybrid assay to determine if the constitutively active and dominant negative forms of MgRac1 interact with Nox1 and Nox2. The results showed that both Nox1 and Nox2 were able to interact with the constitutively active, but not the dominant negative form of MgRac1 (Figure 8A), indicating that Nox1 and Nox2 are indeed MgRac1 effectors.

To determine the effects of deletion and dominant mutations of MgRac1 on ROS production during mycelial and conidial differentiation, we determined NBT content in vegetative hyphae and conidia of the *ΔMgrac1-19*, *MgRac1-CA* and *MgRac1-DN* mutants, and compared with the wild-type strain 70-15. In support of the contention that the Nox proteins are MgRac1 effectors, there was a strong increase in superoxide production in the hyphal tips of the *MgRac1-CA* mutant, while there was a significant decrease in the *ΔMgrac1-19* and *MgRac1-DN* mutants, as quantified by a reduction in the mean pixel intensity due to the accumulation of localized formazan precipitates [34] (Figure 8B and 8C). These results are consistent with the real time PCR data in which the Nox genes are up-regulated in the *MgRac1-CA* mutant but down-regulated in the *ΔMgrac1-19* and *MgRac1-DN* mutants (Table 3). Superoxide production in the *MgRac1* complement strain *Mgrac1-Com* was similar to that of 70-15 in both hyphae and conidia (Figure 8B, 8C, and 8D), indicating full recovery of superoxide production. Interestingly, all mutants including *MgRac1-CA* generated significantly less superoxide than 70-15 in conidia (Figure 8B and 8D), even though *MgRac1-CA* produced more superoxide in hyphae (Figure 8B and 8C). At present, it is unclear why Nox activity undergoes such dramatic changes in hyphae and conidia of the *MgRac1-CA* mutant, but the fact that the conidia derived from the *MgRac1-CA* mutant are nonpathogenic is consistent with a previous report on *Nox* deletion mutants, which also produce nonpathogenic conidia [34].

**Figure 9:**
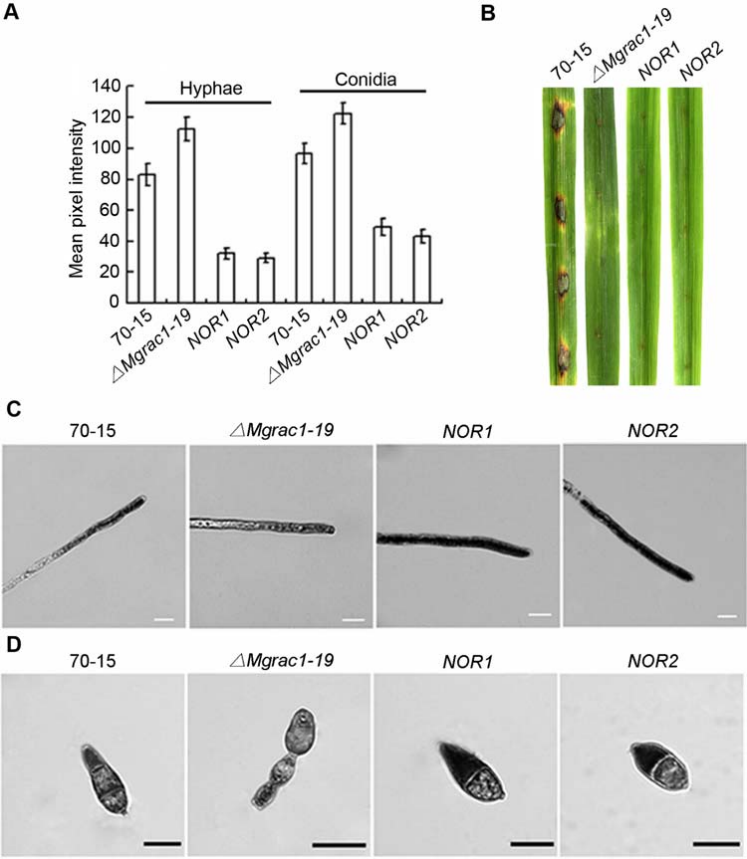
Superoxide production and pathogenicity of *Nox* over-expression mutants. (A) Bar chart showing mean pixel intensity in hyphal tips and conidia of 70-15 (wild-type strain), *ΔMgrac1-19* (the Mgrac1 deletion mutant), *NOR1* (*NOX1* over-expressed in *ΔMgrac1-19*), and *NOR2* (*NOX2* over-expressed in *ΔMgrac1-19*). Error bar means standard deviation based on the data of three independent experiments. Superoxide production was detected by NBT staining. (B) Disease symptoms on the wounded leaf tissues of rice inoculated with mycelial plugs from 70-15, *ΔMgrac1-19*, *NOR1*, and *NOR2*. (C) Detection of superoxide by 0.6 mM NBT staining in the hyphal tips of *Nox* over-expression mutants. Bar = 10 µm. (D) Detection of superoxide by 0.3 mM NBT staining in the conidia of *Nox* over-expression mutants. Bar = 10 µm.

Further epistasis analysis was conducted by over-expression of Nox1 or Nox2 in the *ΔMgrac1-19* mutant. NBT staining showed increased superoxide production in both conidia and mycelia of the over-expression mutants (Figure 9A, 9C, and 9D). However, over-expression of Nox1 or Nox2 in the *ΔMgrac1-19* mutant did not rescue the defect of conidiation (data not shown) and pathogenicity (Figure 9B), even though there was partial recovery in conidial morphology (Figure 9D).

## Discussion

The rice blast fungus *M. grisea* is an important pathogen, causing rice blast disease in a staple food for half of the world's population [10]. In this study, we show that the Rac1 GTPase plays a critical role in the formation of conidia and appressoria for infection of rice. *M. grisea* contains one copy of the Rac1 gene (termed MgRac1), which is highly homologous to its mammalian counterpart [2]. We generated *Mgrac1* deletion mutants of *M. grisea* and found that they have severe defect in conidial production. Of the few conidia formed, most are malformed, elongated, and fail to form appressoria. Consequently the *Mgrac1* deletion mutants cannot effectively infect rice leaves and roots, leading to loss of pathogenicity. Furthermore, we generated *M. grisea* transformants that express dominant negative and constitutively active MgRac1 mutants (*MgRac1-DN* and *MgRac1-CA*). In support of the data on *Mgrac1* deletion mutants, the dominant negative transformant is also defective in the formation of conidia and appressoria and is nonpathogenic. The constitutively active transformant, on the other hand, produces more conidia, with some enlarged than *DN* mutants. Although these conidia can germinate normally, they are also defective in further development into appressorium for infection of rice leaves and onion epidermis.

Rac1 is a member of the Rho GTPase family and generally functions in actin cytoskeleton organization and polarized cell growth [16], which plays an important role in many developmental pathways of diverse organisms. Indeed in the filamentous fungus *P. marneffei*, the Rac homolog CflB is required for cell polarization during asexual development, conidiation and hyphal growth [20]. In the phytopathogenic fungus *U. maydis*, Rac1 is essential for pathogenicity [24]. These observations are consistent with our findings that MgRac1 is essential in *M. grisea* development and pathogenicity. In addition to *M. grisea*, other plant-infecting ascomycetes such as *C. trifolii*, *F. graminearum*, and *S. nodorum* all contain Rac homologs. Our data indicate that MgRac1 plays a critical role in the life cycle of *M. grisea*, specifically in the development of normal infectious structures that allow successful penetration and initiation of plant infection and disease epidemics.

We further identified a Rac1 signaling pathway required for MgRac1-mediated conidiation during the development of *M. grisea*. In this pathway, active, GTP-bound MgRac1 interacts with Chm1 via its PBD domain, leading to the activation of Chm1 kinase activity that could subsequently regulate actin organization and polarized cell growth during the conidiogenesis process. We provide several lines of evidence to support that Chm1 is a major effector of MgRac1 for conidiogenesis in *M. grisea*. First, constitutively active Chm1 corrects the defect of *Mgrac1* deletion mutants in conidiogenesis in terms of morphology and quantity of conidia. However, it cannot correct the defect in appressorial formation and pathogenicity, suggesting that these processes require additional MgRac1 effectors. Second, constitutively active MgRac1 cannot rescue the defect of *chm1* deletion mutants, indicating that Chm1 functions downstream of MgRac1 in the regulation of conidiogenesis. Chm1 is a homolog of mammalian p21-activated kinase (PAK), which is known to interact with and phosphorylate downstream proteins involved in actin cytoskeleton organization and polarized cell growth in mammalian cells [31]. In the dimorphic human pathogenic fungus *P. marneffei*, PAK is required for conidial germination [35]. In the ergot fungus *Claviceps purpurea*, Rac1 and its downstream effector Cla4 function in fungal ROS homoeostasis which could contribute to their drastic impact on differentiation [25]. Cla4 also works as Rac1 downstream effector essential for Rac1-induced filament formation in *U. maydis* [24]. The importance of the MgRac1-Chm1 signaling pathway in the conidiogenesis of *M. grisea* reflects an evolutionarily conserved Rac1 pathway that controls various developmental processes across species via regulation of actin organization and polarized cell growth.

Chm1 is also an effector for Cdc42 in *M. grisea* as shown in the yeast two-hybrid assay (data not shown). Our real-time PCR analysis reveals a potential antagonistic interaction between Rac1 and Cdc42 in *M. grisea*. There is an increase in *Cdc42* expression in *ΔMgrac1-19* and *MgRac1-DN* mutants, while there is a small decrease in *Cdc42* expression in the *MgRac1-CA* mutant (Table 3). However, the conidiogenesis defect in *ΔMgrac1-19* and *MgRac1-DN* mutants is unlikely due to hyperactive Cdc42, because over-expression of Cdc42 has no effect on conidiogenesis (data not shown).

The MgRac1-Chm1 pathway, however, is not sufficient for pathogenesis. Although constitutively active *CHM1ΔPBD* mutant can rescue the conidiation defect of the *Mgrac1* deletion mutant, the resulting conidia remain nonpathogenic, suggesting the involvement of additional effectors, such as the Nox proteins that are NADPH oxidases responsible for ROS production. The *nox1* and *nox2* deletion mutants of *M. grisea* are known to be defective in pathogenesis [34]. In the current study, we show that MgRac1-CA but not MgRac1-DN interacts with Nox1 and Nox2 and promotes superoxide production in *M. grisea*, thus confirming that they are MgRac1 effectors. Consistently, we find that Nox activity is up-regulated in the hyphal tips of the *MgRac1-CA* mutant and down-regulated in the *MgRac1-DN* mutant.

The data from real time PCR, yeast two-hybrid assay and epistasis analysis indicate that Nox1 and Nox2 act as downstream effectors of MgRac1. Although the Nox proteins are required for pathogenesis [34], our data indicate that MgRac1-Nox interaction is not required in conidiation. Unlike Chm1, over-expression of Nox1 or Nox2 cannot rescue the conidiation defect of the *Mgrac1* deletion mutants. Thus, the two MgRac1 signaling pathways play distinct roles in *M. grisea* differentiation, with MgRac1-Chm1 interaction specifically controlling conidiogenesis.

## Materials and Methods

### Fungal strains and growth conditions

*Magnaporthe grisea* (Herbert) Barr parent strains (70-15 and Guy11) and other derivative strains described in this paper were maintained and cultured on the complete medium plates (CM: 0.6% yeast extract, 0.6% casein hydrolysate, 1% sucrose, 1.5% agar) at 25°C. Cultures for genomic DNA isolation, RNA isolation and protoplast preparation were grown in the liquid starch yeast medium (SYM: 0.2% yeast extract, 1% starch, 0.3% sucrose) in a 150-rpm shaker at 25°C for 3–4 d. Conidia were prepared from 10-day-old cultures grown on the oatmeal agar medium (5% oatmeal, 2% sucrose, 1.5% agar) and rice-polish agar medium (2% rice-polish, 1.5% agar, pH 6.0). The selective top agar medium was supplemented with either 400 µg/ml of hygromycin B (Roche Applied Science) or 300 µg/ml of glufosinate ammonium (Sigma-Aldrich Co.), depending on the selection marker in the plasmid vector. Mono-conidial isolation and measurement of conidiation and growth rate were performed as previously described [36].

### Isolation of MgRac1 gene and cDNA

Two PCR primers 1F and 1R (Table 4) were designed based on *Magnaporthe grisea* genome database (www.broad.mit.edu/annotation/genome/magnaporthe.grisea). The *MgRac1* gene was amplified from the 70-15 genomic DNA by a 30-cycle PCR reaction (94°C, 1 min; 54°C, 1 min; 72°C, 1 min), followed by 7 min extension at 72°C. PCR products were cloned into the pGEM-T easy vector (Promega Corp.) and confirmed by direct DNA sequencing. The cDNA of MgRac1 was isolated by RT-PCR of total RNA of *M. grisea* with primers 1F and 1R, followed by cloning into the pGEM-T easy vector and direct DNA sequencing (EF060241).

**Table 4:** Primers used in this study.

Name	Sequence (5′→3′)
1F	TAGGATCCATGGCCGCCCCTGGGGTTC
1R	CGGGATCCTCACAGAATGGTGCACTTTG
2F	TACTCGAGCTTTCTCCGGTCTGGTATATC
2R	GCCTCGAGGGTCTGGAAATATAAGAATGTG
3F	GCAAGCTTAACTACTCGGCTAGTGTTATG
3R	ATGAGCTCCACCGTTACCCTGTGTTGC
4F	CTCGACCCTTCTTGGAGTGG
4R	GACAGACGTCGCGGTGAGTT
5F	TAGGATCCGTTCCAACCTGCGTTGCAAC
5R	CTGAATTCGAGTGTTCAGAGACAGGATG
6F	ATGGCCGCCCCTGGGGTTCAGTCTTTGA
	AGTGTGTCGTCACTGGCGACGTTGCTG
6R	TCACAGAATGGTGCACTTTGACTTC
7F	GAACCAAGCTCGCTCTTCGTGAAGACCCCTC
7R	GAGGGGTCTTCACGAAGAGCGAGCTTGGTTC
8F	GTCGAGCTCCGAAACTTTCCCAAACCGG
8R	TAAGAGCTCAGAAAGACCGGCTGAGTCC
9F	GGTGCCCTGGTATGGGAGACCTCACCAAGCGTAACG
9R	CGTTACGCTTGGTGAGGTCTCCCATACCAGGGCACC
10F	ACGCTGAACCACGCTGAACCATG
10R	GTCGAATCTGTCATGGTGCGAAG
11F	GAAAAGATTCAGAAGACGGAATC
11R	CGCAGATAAGTGCCTGGTCGTAC
12F	GCTGTCCTCGTCGATCTCGA
12R	CAGAGCAGGTCAGGTAACGA
13F	CGGAATTCATGGCCGCCCCTGGGG
13R	GCGGATCCTCACAGAATGGTGGACTTTG
14F	CACGAATTCATGAACCCTGGACCTGCC
14R	TAAGAGCTCTTATTTGGCATGCTTCTTGAAGG
15F	AGACGAAGAAGCCGATAGCAC
15R	CGGTTTCCGACATGGTTGAC
16F	CGTTCGGCACCTTACACGA
16R	CCCTCCGCTGGTTCACCAA
17F	GTGTGTCGTCACTGGCGA
17R	ACTGTGGGGATGTACTCGC
18F	ATGATCGGTGACGAGCCGT
18R	GTATGATAGGGGTCGCAGC
19F	GAGACTTGTCAGGGACTG
19R	TGACGTTACCCCTGGCAT
20F	CAACTTCTTCAATGTCGAG
20R	AAGCATACACAACAGCATC
21F	ATTGCCAGAGCTGCGGCG
21R	AGGCGTTTGACGCGCAAGA
22F	TCCGTGGAAAGGTTTCCATG
22R	ATCCACTCGACGAAGTACGA
23F	CCCATCGATACATGTCGGTCGGAGAGTTCTTG
23R	CTCGGATCCCTAGAAATGCTCCTTCCAGAAG
24F	TACGAATTCATGTCTGGATACGGCTACGG
24R	TGTGGATCCCTAGAAATTCTCCTTGCCCC
25F	TATCTCGAGATAAATGTAGGTATTACCTGTAC
25R	GATGGATCCTTTGAAGATTGGGTTCCTAC
26F	TATGGATCCATGTCGGTCGGAGAGTTCTTGG
26R	CGGACGCGTCTAGAAATGCTCCTTCCAGAAGCGG
27F	TATGGATCCATGTCTGGATACGGCTACGG
27R	GAAACGCGTCTAGAAATTCTCCTTGCCCC

### MgRac1 gene replacement and mutants

To replace the gene, a 0.9-kb fragment upstream of the *MgRac1* ORF in the *M. grisea* genome was amplified with primers 2F and 2R (Table 4) and cloned into the *Xho*I sites on pCSN43, and the resulting construct is named pRAC11. Then a 1.0-kb fragment downstream of *MgRac1* ORF was amplified with primers 3F and 3R (Table 4) and cloned between the *Hin*dIII and *Sac*I sites in pRAC11, and the resulting construct was the *MgRac1* gene replacement vector, pKRA1, which had the selective marker *hph* gene flanked by the MgRac1 ORF flanking sequences. pKRA1 was then transformed into protoplasts of the wild-type strain 70-15 as described previously [37]. Hygromycin-resistant transformants were screened by PCR with primers 4F and 4R (Figure 1A, Table 4) to confirm that the MgRac1 gene was deleted. These transformants were *Mgrac1* deletion mutants.

The complementation vector pCRA1 was constructed by cloning a 2.37-kb fragment containing the native promoter and ORF of *MgRac1*, amplified by PCR with primers 5F and 5R (Table 4), into the basta-resistance vector pBARKS1. The complementary strain *Mgrac1*-Com was generated by reintroduction of pCRA1 into the *Mgrac1* deletion mutants, followed by screening for basta-resistant transformants and PCR confirmation.

The constitutively active and dominant negative MgRac1 mutants (*MgRac1-CA* and *MgRac1-DN*) were generated by site-directed mutagenesis of wild type MgRac1 via a PCR-based approach. Two primers including the forward primer 6F and reverse primer 6R (Table 4) were used to generate *MgRac1-CA* with 6F containing the substitution of the glycine (G17) of MgRac1 with valine. The dominant negative MgRac1 mutant (*MgRac1-DN*) was generated by substitution of the aspartic acid (D123) with alanine by recombinant PCR with two pairs of primers 1F/7R and 7F/1R, with 7F and 7R containing the mutation (Table 4). Wild type MgRac1 cDNA was amplified with primers 1F and 1R (Table 4) to construct over-expression MgRac1 mutant. All the mutated and wild-type DNA fragments were amplified with pfu polymerase (Stratagene), confirmed by DNA sequencing, and cloned into the vector pTE11. The expression of *MgRac1-CA*, *MgRac1-DN* and *MgRac1-OE* was driven by the constitutive RP27 promoter built within pTE11, upon transformation of protoplasts of the wild-type strain 70-15, the *chm1* deletion mutant and the Guy11 strain expressing the heterologous *Aspergillus nidulans* tropomyosin-GFP [29].

### *CHM1ΔPBD* mutants and Nox over-expression mutants

To generate the *CHM1ΔPBD* (deletion of the PBD domain^185–243^ in the *Chm1* ORF) construct, the genomic DNA of wild-type strain 70-15 was amplified by recombinant PCR with four primers 8F/9R and 9F/8R (Table 4). The resulting PCR product contained the CHM1*ΔPBD* sequence driven by the native *Chm1* promoter. It was then digested with *Sac*I and cloned into pBARKS1, resulting in the CHM1*ΔPBD* expression vector pBCP17. After transforming the wild-type strain Guy11 and *Mgrac1* deletion mutant with pBCP17, basta-resistant transformants were isolated and screened by PCR with primers 8F and 8R to confirm the *CHM1ΔPBD* sequence. The expression of *CHM1ΔPBD* in these transformants was confirmed by Northern blot analysis (see below).

*M. grisea* Nox1 and Nox2 cDNAs were amplified by RT-PCR with primers 26F/26R and 27F/27R (Table 4) and cloned into the *Xho*I/*Bam*HI sites of pKNTP vector, which contained the constitutive RP27 promoter and the neomycin gene as a selection marker. The pKNTP vector was derived from pKNTG via insertion of the RP27 promoter, which was amplified from pTE11 by PCR with the primers 25F and 25R (Table 4). The resulting Nox1 and Nox2 expressing constructs were termed pOENO1 and pOENO2, respectively. Upon transformation of *Mgrac1* deletion mutants with pOENO1 or pOENO2, 300 µg/ml of neomycin sulfate (Amresco Inc.) was supplemented for selection. Neomycin-resistant transformants were screened and Nox expression was confirmed by NBT staining.

### Southern blot and Northern blot analysis

For Southern blot analysis, genomic DNA was isolated from *M. grisea* wild-type strain 70-15, putative *Mgrac1* deletion mutants and ectopic transformants, following the miniprep procedure [37]. DNA aliquots of 5 µg were digested with *Pst*I, separated by electrophoresis on 1% agarose gels and transferred onto a Hybond N+ membrane (Amersham Pharmacia Biotech). Interior probe was amplified with primers 10F and 10R (Figure 1A, Table 4), while exterior probe was amplified with primers 11F and 11R (Figure 1A, Table 4).

For Northern blot analysis, total RNA samples (10 µg per sample), which were isolated from growing hyphae of *M. grisea* using the RNAiso Reagent (Takara Bio Inc.), were separated by electrophoresis on 1% formaldehyde denaturing gel and transferred onto a Hybond N+ membrane (Amersham Pharmacia Biotech). The probe for Northern hybridization was the 0.5-kb *Chm1* exon region amplified by primers 15F and 15R (Table 4). For internal control, a 0.73-kb PCR fragment for 18s rRNA (AB026819) was amplified from *M. grisea* genomic DNA using primers 16F and 16R (Table 4).

For both Southern and Northern blot analysis, probe labeling, hybridization and detection were performed with DIG High Prime DNA Labeling and Detection Starter Kit I (Roche Applied Science), following the manufacturer's instructions.

### RT-PCR and real-time PCR analysis

First strand cDNA was synthesized with the ImProm-II Reverse Transcription System (Promega Corp.) following the manufacturer's instructions. For RT-PCR, a 2 µl aliquot of first-strand cDNA was subjected to 30 cycles of PCR amplification with *MgRac1* ORF primers 1F and 1R. The amount of template cDNA was normalized by PCR with a pair of β-tubulin (XP_368640) primers 12F and 12R (Table 4). Twelve microliters of PCR products were analyzed by 1.5% agarose gel electrophoresis.

In quantitative real-time PCR, *MgRac1*, *MgCdc42* (AF250928), *Chm1* (AY057371), *Nox1* (EF667340) and *Nox2* (EF667341) were amplified by the following pairs of primers: 17F/17R, 18F/18R, 19F/19R, 20F/20R, and 21F/21R, respectively (Table 4). As an endogenous control, an 86-bp amplicon of β-tubulin gene was amplified with primers 22F and 22R (Table 4). Quantitative real-time PCR was performed with the MJ Research OPTICON Real-Time Detection System using TaKaRa SYBR Premix Ex Taq (Perfect Real Time) (Takara, Japan). The relative quantification of the transcripts was calculated by the 2^−ΔΔCt^ method [38].

### Analysis of conidial morphology, conidial germination, appressorial formation and penetration

Conidia were prepared from 10-day-old oatmeal agar cultures. For the measurement of the length and width of conidia, five independent experiments were performed with 3 replicates each time, and 50 conidia were observed in each replicate. Mean and standard deviation were calculated using SPSS V13.0, and one way ANOVA was performed on the data for significant differences between genotypes. Aliquots (50 µl) of conidial suspensions (5×10^4^ conidia/ml) were applied on the hydrophobic side of Gelbond film (Cambrex BioScience). The conidial droplets were incubated in a moist chamber at 25°C. Conidial germination and appressorial formation were examined at 0.5, 1, 2, 4, 8 and 24 h post-incubation. Appressorial penetration on onion epidermal strips was assayed as described previously [39]. Photographs were taken with an Olympus BX51 universal research microscope.

### Plant infection assay

Rice (*Oryza sativa* L.) and barley (*Hordeum vulgare* cv. Jinchang 1316) seedlings (15 and 8-day-old respectively) were grown under the conditions described previously [36]. The rice cultivar used for infection assays was CO39 [40]. Conidial suspensions (1×10^5^ conidia/ml in 0.02% Tween solution) were prepared from oatmeal agar cultures for spray or wounded infection assays. Plant incubation and inoculation were performed as described [5]. Root infection assays were carried out as described [41]. Lesion formation was examined at 7 days after inoculation on rice and 5 days after inoculation on barley. The mean of lesion numbers formed on 5-cm leaf tips was determined as described previously [42],[43]. Cell walls and septa of vegetative hyphae were visualized by Calcofluor White (10 µg/ml, Sigma), and nuclei of vegetative hyphae were visualized by DAPI (50 mg/ml, Sigma) as described [44].

### Yeast two-hybrid assay

The MATCHMAKER GAL4 Two-Hybrid System 3 (Clontech) was used to determine protein–protein interactions. The *MgRac1* cDNA was amplified with primers 13F and 13R (Table 4) and inserted into the *Eco*RI and *Bam*HI sites of the yeast vector pGBKT7 (Clontech). MgRac1 contains the C-terminal CAAL motif that is subject to prenylation at the cysteine residue. This modification makes these Rho-family GTPases membrane associated and difficult to enter the nucleus for protein interactions in the two-hybrid assay. Thus, we constructed MgRac1:C196S mutants that cannot be prenylated and is thus soluble. Constitutively active and dominant negative mutations were generated at the MgRac1:C196S background and the resulting double mutants were used as the baits in the two-hybrid assay. *Chm1* ORF was amplified with primers 14F and 14R (Table 4) and cloned between the *Eco*RI and *Sac*I sites on the yeast vector pGADT7 (Clontech) as the prey in the two-hybrid assay. The *CHM1ΔPBD* cDNA was amplified by recombinant PCR with two pairs of primers (14F/9R and 9F/14R) from the first-strand cDNA of wild-type 70-15, followed by cloning into the *Eco*RI and *Sac*I sites of pGADT7 as a prey in the two-hybrid assay. *Nox1* and *Nox2* ORFs were amplified with primers 23F/23R and 24F/24R, respectively (Table 4), and cloned into the yeast vector pGADT7 (Clontech) as the preys in the two-hybrid assay. The resulting bait and prey vectors confirmed by sequencing were co-transformed in pairs into the yeast strain AH109 (Clontech). The Leu^+^ and Trp^+^ transformants were isolated and assayed by X-gal staining. Positive clones were further confirmed by plating onto SD-Leu-Trp-His media for the *HIS3* reporter gene expression.

In all assays, the interaction of pGBKT7-53 and pGADT7-T was used as the positive control, and the interaction of pGBKT7-Lam and pGADT7-T as the negative control.

### In vitro PAK kinase assay

Vegetative hyphae were harvested from 3-day-old CM liquid cultures for protein isolation. About 200 mg of mycelia were resuspended in 2 ml of extraction buffer (50 mM Tris-HCl [pH 7.5], 100 mM NaCl, 50 mM NaF, 2 mM phenylmethylsulfonyl fluoride, 5 mM EDTA, 1 mM EGTA, 1% Triton X-100, 10% glycerol) and centrifuged. Protein concentration was measured by GeneQuant pro spectrophotometer (Amersham Biosciences), and 10 µg of total protein was applied for kinase activity detection. PAK Kinase assay was performed by using the HTScan PAK1 kinase assay kit, according to the manufacturer's instructions (Cell Signaling Technology).

### ROS detection assay

For superoxide detection, hyphae of wild-type strain 70-15 and *MgRac1* mutants were collected from 3-day CM agar plates and stained with 0.6 mM NBT (nitroblue tetrazolium) aqueous solution for 2 h. Superoxide production in the hyphal tips was viewed by bright-field microscopy. Conidia were collected from 10-day oatmeal agar plates and stained with 0.3 mM NBT aqueous solution for 1 h. After incubation in NBT, the reaction was stopped by the addition of ethanol, and the pattern of formazan staining was observed by using Zeiss Axiovert 200 M microscope equipped with a Zeiss LSM 510 META system. The intensity of formazan precipitation in conidia and hyphal tips was quantified by using Meta Imaging Series 6.1 software (Universal Imaging Corporation) to calculate mean pixel intensity within regions of interest fitted to the outline structure. Measurements were made on the most intensely stained conidia and hyphae of each strain. Pixel intensity was reduced in areas of formazan precipitation.

### Gene accession numbers

GenBank accession numbers for genes or proteins used in this article are EF060241 (MgRac1), AF250928 (MgCdc42), AY057371 (Chm1), EF667340 (Nox1), EF667341 (Nox2), XP_368640 (β-tubulin) and AB026819 (18s rRNA).
